# Hepatic AhR Activation by TCDD Induces Obesity and Steatosis via Hepatic Plasminogen Activator Inhibitor-1 (PAI-1)

**DOI:** 10.3390/ijms26178452

**Published:** 2025-08-30

**Authors:** Seung Jun Oh, Suyeol Im, Sora Kang, Aden Geonhee Lee, Byung Cheol Lee, Youngmi Kim Pak

**Affiliations:** 1 Department of Biomedical Sciences, Graduate School, Kyung Hee University, Seoul 02447, Republic of Korea; ohsungjun124@khu.ac.kr (S.J.O.); suryeol@khu.ac.kr (S.I.); 2 Biomedical Science Institute, Kyung Hee University, Seoul 02447, Republic of Korea; ksr8947@khu.ac.kr; 3 Department of Physiology, School of Medicine, Kyung Hee University, Seoul 02447, Republic of Korea; al2592@cornell.edu; 4 Department of Nutritional Sciences, College of Human Ecology, Cornell University, Ithaca, NY 14853, USA; 5 Department of Oriental Internal Medicine, Kyung Hee University, Seoul 02447, Republic of Korea

**Keywords:** obesity, aryl hydrocarbon receptor, 2,3,7,8-Tetrachlorodibenzodioxin, lipid accumulation, inflammation, mitochondria, PAI-1

## Abstract

Exposure to persistent organic pollutants such as 2,3,7,8-tetrachlorodibenzodioxin (TCDD) increases metabolic disorder risk. In this study, we show that a single intraperitoneal injection of TCDD (10 μg/kg) in C57BL/6J mice induced body weight gain, lipid accumulation in the liver and adipose tissue, macrophage infiltration, and elevated hepatic and serum triglyceride levels after 12 weeks. Despite serum aryl hydrocarbon receptor (AhR) ligand levels normalizing by 12 weeks, the persistent effects suggest TCDD sequestration in fat tissue. TCDD inhibited the expression of mitochondrial proteins (COX1, TOM20, TFAM, H2AX) and reduced mitochondrial oxygen consumption. Liver-specific AhR knockout ameliorated TCDD-induced mitochondrial dysfunction, lipid accumulation, and macrophage infiltration. Mechanistically, TCDD-induced hepatic plasminogen activator inhibitor-1 (PAI-1) promoted adipocyte hypertrophy. In the liver, PAI-1 disrupted the interaction between tissue-type plasminogen activator (tPA) and apolipoprotein B (ApoB), thereby enhancing very-low-density lipoprotein (VLDL) assembly. These findings reveal that hepatocyte-derived circulating PAI-1, upregulated via hepatic AhR activation, contributes to adipocyte hypertrophy and hepatosteatosis through the intracellular modulation of the tPA–PAI-1 axis. Thus, hepatic AhR activation drives mitochondrial dysfunction and obesity, even after a single TCDD exposure.

## 1. Introduction

Obesity is characterized by the excessive accumulation of fat in the body and is defined by a body mass index (BMI) greater than 30 kg/m^2^. According to the World Health Organization (WHO), in 2022, 1 in 8 people in the world were living with obesity. Since 1990, worldwide adult obesity has more than doubled, and adolescent obesity has quadrupled. The excessive fat deposits are thought to be caused by increased energy intake and decreased physical activity. However, an energy imbalance alone is not enough to explain the rising incidence of obesity, as it is influenced by complex contributors including genetic, metabolic, psychological, and environmental factors [1,2]. In addition, various epidemiological studies have reported the potential of environmental chemicals to increase the risk of overweight and their obesogenic effects [3,4,5,6].

Persistent organic pollutants (POPs), such as dioxins, polychlorinated biphenyls, and organochlorines, are synthetic chemicals, which are produced during industrial processes. POPs are lipophilic and not readily degradable in nature, leading to their accumulation in the environment and human tissues through the consumption of contaminated food or air pollution. Dioxins constitute a family of 210 structurally related congeners, among which 2,3,7,8-tetrachlorodibenzo-p-dioxin (TCDD) is recognized as the most toxic. The acute toxicity of TCDD at high doses was strikingly demonstrated in the aftermath of the Seveso industrial accident in Italy in 1976 [7]. Today, however, TCDD is ubiquitously present in the environment at low concentrations, particularly in industrialized regions, largely as a consequence of anthropogenic activities [8]. Although the use of chlorine-based industrial processes has declined, uncontrolled combustion events—including waste incineration, wildfires, and volcanic activity—have emerged as major contemporary sources of dioxin emissions. Following the adoption of the Stockholm Convention in 2001 and the implementation of regulatory measures aimed at reducing dioxin emissions across Europe, sediment core analyses have revealed a downward trend in environmental dioxin levels [9]. Nevertheless, considerable regional variability in dioxin contamination persists, underscoring the need for continued monitoring and mitigation efforts [10].

Aryl hydrocarbon receptor (AhR) is a potent biological receptor for exogenous chemicals [11]. Dioxin acts as a representative ligand for AhR. Binding of AhR with its ligands in cytosol leads to the transcription of genes involved in various metabolisms including xenobiotics, inflammation, and lipid metabolism [12,13,14]. Interestingly, Sprague-Dawley rats fed a high-fat diet containing crude fish oil from Atlantic salmon showed exacerbated insulin resistance, obesity, and hepatosteatosis compared to rats fed a high-fat diet with refined fish oil, indicating that POPs commonly present in food chains lead to metabolic disorders [15]. In addition, HBU651, a novel AhR antagonist, ameliorated high-fat diet-induced inflammation and obesity in mice, suggesting the involvement of AhR in the incidence of obesity [16].

Oxidative phosphorylation (OXPHOS) complexes are responsible for ATP synthesis in mitochondrial inner membrane. Translocase of outer mitochondrial membrane 20 (TOM20), located in the outer mitochondrial membrane, plays a crucial role in importing cytosolic proteins into mitochondria. TOM20 recognizes mitochondria-targeting sequences in precursor proteins and guides them to the TOM complex for import. Additionally, mitochondria possess their own genome, mitochondrial DNA, responsible for synthesizing mitochondrial proteins. Mitochondrial transcription factor A (TFAM) binds to mitochondrial DNA and regulates its transcription and maintenance. Abnormal mitochondrial function is an important feature of steatohepatitis, and fatty liver disease is strongly and intricately associated with obesity and type 2 diabetes [17,18]. Obese humans with nonalcoholic steatohepatitis exhibited impaired mitochondrial respiration and biogenesis, and elevated oxidative stress [19].

TCDD exerts a broad spectrum of biological effects by disrupting homeostasis through its actions on the immune, nervous, and reproductive systems, as well as by altering the function of multiple organs, including the skin, liver, pancreas, and adipose tissue. The International Agency for Research on Cancer (IARC) has classified TCDD as a Group 1 human carcinogen, underscoring its toxicological significance. In the context of energy metabolism, environmental chemicals such as endocrine-disrupting compounds (EDCs) have been implicated in the pathogenesis of obesity. These agents, often termed “obesogens,” are thought to contribute to metabolic dysregulation by impairing lipid metabolism and promoting adipogenesis, thereby exacerbating the development of obesity [20]. Studies have reported that chronic exposure to TCDD elicits hepatic lipid accumulation, inflammation, and fibrosis [12,21,22]. TCDD-induced intracellular ROS generation and mitochondrial dysfunction [23], and repressed gene expression related to β-oxidation [24]. POPs in plasma from elderly individuals were associated with increased oxidative stress [25]. Furthermore, elevated circulating AhR ligands were related to the prevalence of diabetes and showed mitochondria-inhibiting activity in humans [24,26].

Several previous DNA microarray reports documented that plasminogen activator inhibitor-1 (PAI-1) expression could be induced by TCDD [27]. Recent studies have revealed a novel role for the fibrinolytic system in hepatic lipid metabolism, particularly through the interaction between PAI-1, tissue plasminogen activator (tPA), and apolipoprotein B (ApoB). Microsomal triglyceride transfer protein (MTP), a rate-limiting enzyme in hepatic very-low-density lipoprotein (VLDL) secretion, plays a critical role in this process by translocating newly synthesized ApoB100 into the endoplasmic reticulum (ER) and facilitating the lipidation of ApoB100 with neutral lipids to form primordial VLDL particles. Under normal conditions, tPA can bind to the N-terminal region of ApoB in hepatocytes, interfering with MTP-mediated lipidation of ApoB and thereby reducing VLDL assembly. Upon TCDD exposure, AhR activation upregulates PAI-1, which then complexes with tPA to dissociate tPA from ApoB. This may promote ApoB lipidation and increases VLDL assembly and secretion [28].

In this study, we monitored the chronic metabolic effects of a single intraperitoneal exposure to TCDD, focusing on lipid accumulation, inflammation, and mitochondrial dysfunction in the liver and adipose tissue of mice fed a standard diet. This experimental paradigm is distinct from conventional models employing repeated high-dose or sub-chronic TCDD exposure. We further examined the role of hepatic AhR was investigated in mediating these effects to better understand how AhR activation contributes to the pathogenesis of metabolic disorders.

## 2. Results

### 2.1. Lipid Accumulation and Downregulation of Mitochondrial Proteins Following a Single TCDD Injection

**Figure 1:**
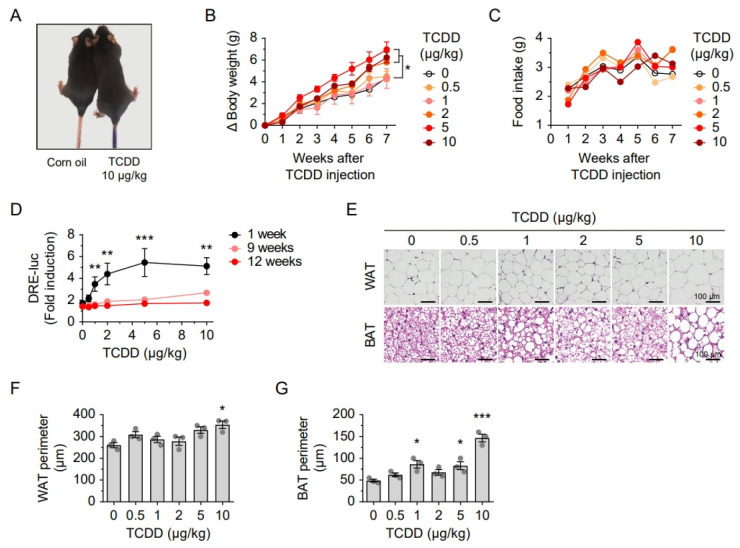
A single injection of TCDD increased body weight and induced adipocyte hypertrophy in mice. (**A**) Representative images of mice 12 weeks after injection with corn oil (left) or 10 μg/kg TCDD (right). (**B**) Body weight changes and (**C**) food intake following injection with various doses of TCDD (0, 0.5, 1, 2, 5, and 10 μg/kg). (**D**) Serum AhR ligand levels measured by luciferase assay at 1, 9, and 12 weeks post-injection. (**E**) Representative H&E-stained images of epididymal white adipose tissue (WAT) and interscapular brown adipose tissue (BAT) 12 weeks after TCDD injection. Scale bars, as indicated. (**F**,**G**) Quantification of WAT (**F**) and BAT (**G**) perimeters in mice injected with TCDD. Data are presented as means ± SEM (n = 7). * *p* < 0.05, ** *p* < 0.01, *** *p* < 0.001 vs. corn oil-injected controls.

To investigate the metabolic effects of TCDD exposure, we administered a single intraperitoneal injection of TCDD at various doses to mice fed a standard diet. Mice injected with TCDD exhibited increased body weight compared to corn oil-injected controls at 12 weeks post-injection (Figure 1A,B). A dose-dependent increase in body weight was observed in mice treated with 2, 5, or 10 μg/kg TCDD, without significant changes in food intake (Figure 1B,C). Serum levels of AhR ligands were assessed using the CALA assay. Luciferase activity, indicating serum AhR ligand levels, increased in a dose-dependent manner at 1-week post-injection but returned to baseline by 9 and 12 weeks (Figure 1D). Despite normalized serum AhR ligand levels at later time points, mice injected with 10 μg/kg TCDD showed significant increases in the perimeters of both white adipose tissue (WAT) and brown adipose tissue (BAT) at 12 weeks post-injection (Figure 1E–G). These results indicate that lipid accumulation and adipocyte hypertrophy persist following TCDD exposure, suggesting that the lipophilic nature of POPs may lead to their sequestration in fat tissue, thereby influencing metabolic homeostasis.

### 2.2. TCDD Promotes Liver Injury and Mitochondria Dysfunction

**Figure 2:**
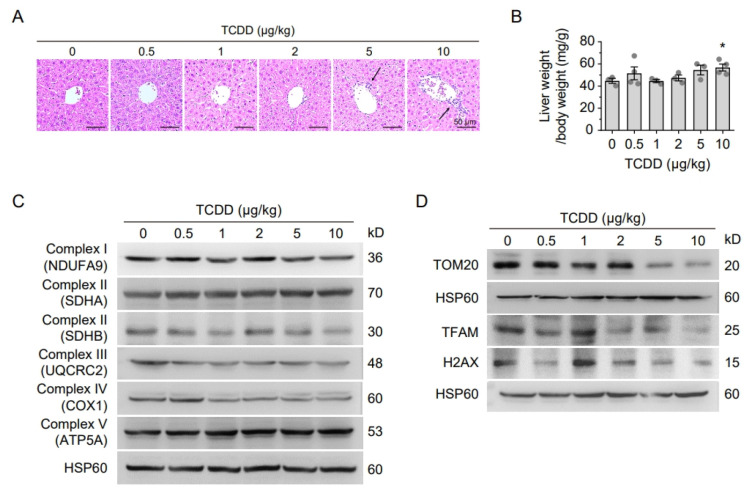
A single TCDD injection induces hepatic inflammation and reduces mitochondrial protein expression. (**A**) H&E-stained liver sections 12 weeks after TCDD injection (0–10 μg/kg). Arrows indicate infiltrating immune cells, increased at higher doses. (**B**) Liver-to-body weight ratio. Mice injected with 10 μg/kg TCDD showed significantly increased liver weight. (**C**) Western blot of mitochondrial OXPHOS complex subunits (NDUFA9, SDHA, SDHB, UQCRC2, COX1, ATP5A). Expression decreased dose-dependently, notably at 5 and 10 μg/kg. (**D**) Western blot analysis of mitochondrial structural and regulatory proteins (TOM20, TFAM, and H2AX) with HSP60 used as a loading control. Expression of TOM20, TFAM, and H2AX was reduced at higher TCDD doses. Data are means ± SEM (n = 5–6). * *p* < 0.05 vs. vehicle control.

Histopathological analysis of H&E-stained liver sections revealed notable infiltration of inflammatory cells in mice injected with 5 or 10 μg/kg TCDD, indicating that even a single moderate-dose exposure elicits an acute hepatic immune response (Figure 2A). Additionally, injection of 10 μg/kg TCDD significantly increased liver weight relative to body weight, suggesting hepatomegaly associated with steatosis or inflammatory injury (Figure 2B). To elucidate the mechanisms underlying these hepatic changes, the expression of mitochondrial proteins related to oxidative phosphorylation and mitochondrial integrity was evaluated. Western blot analyses showed marked downregulation of cytochrome c oxidase subunit 1 (COX1), TOM20, TFAM, and the histone variant H2AX (Figure 2C,D, Supplementary Figures S1 and S2). These reductions indicate that TCDD exposure leads to mitochondrial dysfunction, potentially through suppression of mitochondrial biogenesis, impairment of structural integrity, altered mitochondrial turnover, and/or mitochondrial protein import. The decrease in H2AX may further suggest enhanced miR-24 expression [29] under TCDD-induced stress conditions.

### 2.3. Attenuated Lipid Accumulation and Inflammation in AhR LKO Mice

**Figure 3:**
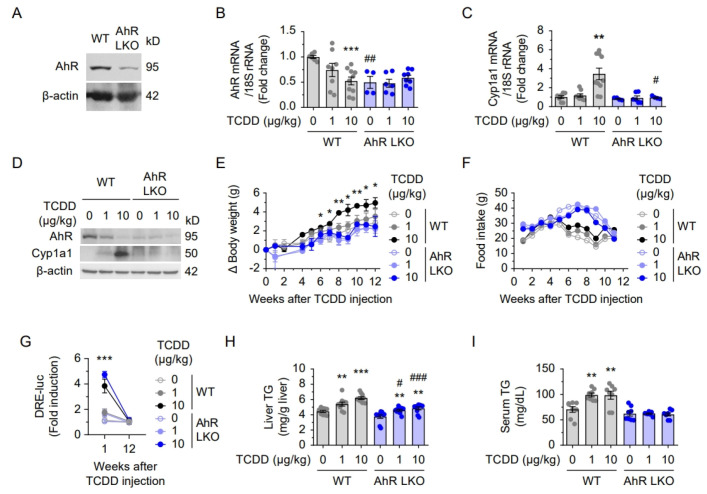
Liver-specific AhR knockout attenuated TCDD-induced lipid accumulation. (**A**) AhR expression in WT and AhR LKO mice. (**B**,**C**) mRNA levels of AhR (**B**) and Cyp1a1 (**C**) after TCDD injection (0, 1, or 10 μg/kg). (**D**) Western blot analysis of AhR and Cyp1a1 in liver tissues. (**E**) Body weight changes and (**F**) food intake after TCDD injection. (**G**) Serum AhR ligand levels measured by CALA luciferase assay. (**H**) Triglyceride (TG) levels in liver lysates and (**I**) serum TG after TCDD injection. Gray and blue circles in bar graphs represent individual data points. Data are presented as means ± SEM (n = 6~7). * *p* < 0.05, ** *p* < 0.01, *** *p* < 0.001 vs. corn oil-injected mice; # *p* < 0.05, ## *p* < 0.01, ### *p* < 0.001 vs. WT mice.

To assess the role of hepatic AhR in TCDD-induced effects, liver-specific AhR knockout (AhR LKO) mice were used (n = 7). To validate AhR deletion, AhR expression was evaluated at both protein and mRNA levels in the liver. Western blot analysis confirmed the loss of hepatic AhR protein in AhR LKO mice, while it was clearly detected in WT controls (Figure 3A). Consistently, while AhR mRNA levels in WT mice decreased upon TCDD exposure due to normal negative feedback, this response was not observed in AhR LKO mice, confirming that hepatic AhR was effectively deleted (Figure 3B). Likewise, Cyp1a1 mRNA, a classical AhR target, was robustly induced by TCDD in WT mice, but this induction was completely abolished in AhR LKO mice (Figure 3C). Western blot also confirms that hepatic AhR was effectively deleted in the LKO model (Figure 3D), validating the successful generation of AhR LKO mice.

Body weight gain was significantly attenuated in AhR LKO mice starting from 6 weeks post-injection, despite their higher food intake (Figure 3E,F). Serum AhR ligand levels increased at 1 week in both WT and AhR LKO mice but returned to baseline by 12 weeks (Figure 3G). TCDD induced substantial hepatic triglyceride (TG) accumulation in the liver and serum of WT mice, whereas TG accumulation was reduced in the liver and absent in the serum of AhR LKO mice (Figure 3H,I), indicating that hepatic AhR is essential for TCDD-induced lipid dysregulation.

### 2.4. Hepatic AhR Deficiency Alleviates TCDD-Induced Lipid Accumulation and Inflammation

**Figure 4:**
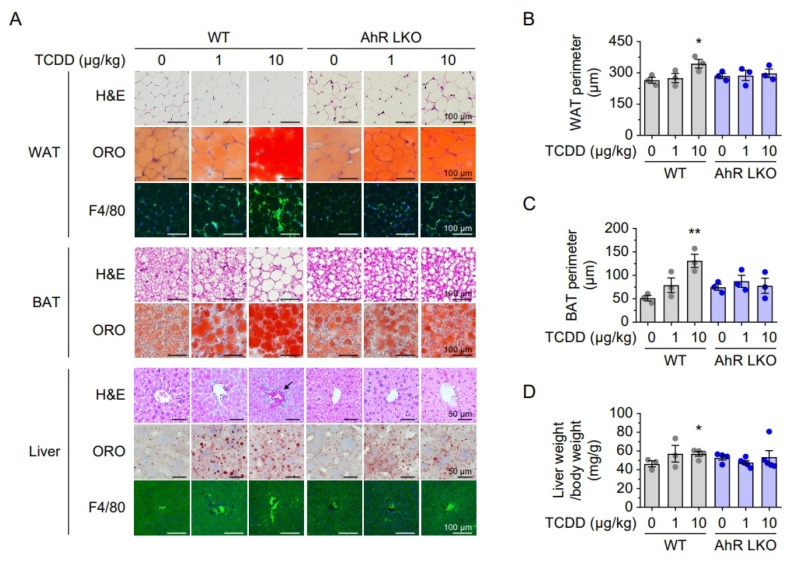
Hepatic AhR regulates lipid accumulation and macrophage infiltration in adipose tissues and liver. (**A**) Histological analyses of WAT, BAT, and liver tissues from WT and AhR LKO mice. Top panels: H&E staining. Black arrow indicates macrophage infiltration. Middle panels: Oil Red O (ORO) staining for lipid accumulation. Bottom panels: immunostaining for F4/80 to detect macrophage infiltration. (**B**) Quantification of adipocyte perimeters in epididymal WAT. (**C**) Quantification of adipocyte perimeters in interscapular BAT. (**D**) Liver weight relative to body weight. Data are presented as means ± SEM (n = 3). * *p* < 0.05, ** *p* < 0.01 vs. corn oil-injected mice.

Histological analyses were performed on WAT, BAT, and liver tissues from WT and AhR LKO mice 12 weeks after a single TCDD injection (0, 1 or 10 μg/kg). In WT mice, TCDD significantly increased adipocyte size in WAT and BAT in a dose-dependent manner, whereas this hypertrophy was attenuated in AhR LKO mice (Figure 4A,C). Oil Red O (ORO) staining revealed marked hepatic lipid accumulation and increased liver-to-body weight ratio in WT mice, while these effects were significantly reduced in AhR LKO mice (Figure 4A,D). Furthermore, F4/80 immunostaining showed increased macrophage infiltration in the WAT and liver of WT mice after TCDD exposure, indicating an inflammatory response. In contrast, this inflammatory cell infiltration was not observed in the corresponding tissues of AhR LKO mice (Figure 4A). These results suggest that hepatic AhR is essential for mediating TCDD-induced adipocyte hypertrophy, hepatic steatosis, and inflammation.

### 2.5. Hepatic AhR-Dependent Mitochondrial Dysfunction After TCDD Injection

**Figure 5:**
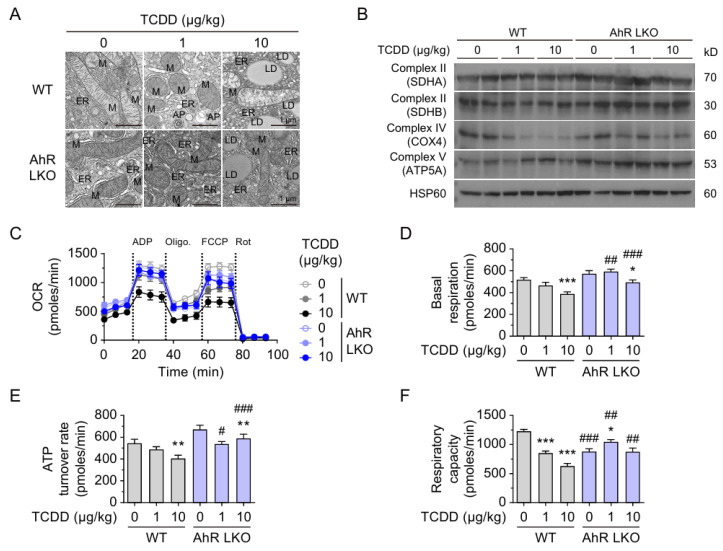
TCDD injection induces mitochondria dysfunction and lipid droplet accumulation. (**A**) Transmission electron microscopy images of liver tissues from WT and AhR LKO mice injected with TCDD (0, 1, or 10 μg/kg). Mitochondria (M), endoplasmic reticulum (ER), lipid droplets (LD), and autophagosomes (AP) are indicated. (**B**) Western blot analysis of OXPHOS complex subunits in mitochondria isolated from liver tissues. (**C**–**F**) Oxygen consumption rate (OCR) measurements in isolated liver mitochondria. (**C**) Representative OCR profiles. (**D**) Basal respiration. (**E**) ATP turnover rate. (**F**) Respiratory capacity. Calculations were performed as described in Methods (n > 20). Data are presented as means ± SEM (n = 6). * *p* < 0.05, ** *p* < 0.01, *** *p* < 0.001 vs. corn oil-injected mice; # *p* < 0.05, ## *p* < 0.01, ### *p* < 0.001 vs. WT mice.

Given the decreased expression of mitochondrial proteins including COX1, TOM20, and TFAM, the ultrastructure and function of liver mitochondria were assessed using electron microscopy and oxygen consumption rate (OCR) measurements. Electron microscopy revealed that TCDD treatment (10 μg/kg) induced ER fragmentation, disrupted mitochondrial cristae, autophagosome formation, and increased lipid droplet formation in the livers of WT mice (Figure 5A). Interestingly, in AhR LKO mice, the ER appeared dilated even without TCDD injection, and lipid droplets were also increased upon TCDD treatment. Western blot analysis showed that COX1 expression was decreased in WT mice after TCDD injection, while its reduction was marginal in AhR LKO mice, suggesting partial AhR dependency (Figure 5B, Supplementary Figures S3 and S4). OCR measurements demonstrated that TCDD reduced basal respiration, ATP turnover, and respiratory capacity in mitochondria isolated from WT livers (Figure 5C–F). In AhR LKO mice, respiratory capacity was reduced at baseline; however, TCDD-induced decreases in basal respiration and ATP turnover were minimal or absent, indicating that hepatic AhR knockout mitigated TCDD-induced mitochondrial dysfunction.

### 2.6. Secreted Hepatic PAI-1 Promotes Adipocyte Hypertrophy

**Figure 6:**
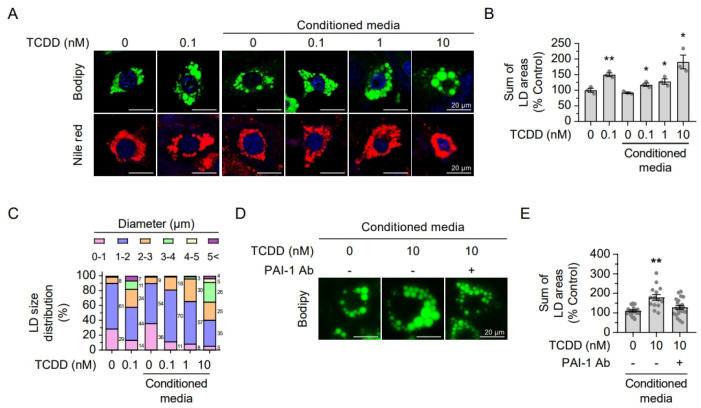
Conditioned media from TCDD-treated Hepa1c1c7 cells promoted adipogenesis in 3T3-L1 cells. (**A**–**C**) Lipid droplet formation after incubation with conditioned media from Hepa1c1c7 cells in 3T3-L1 cells. (**A**) Fluorescence images of lipid droplets (LD) stained with Bodipy (green) or Nile red (red). DAPI (blue) staining for nuclei. (**B**) Sum of LD areas. (**C**) LD size distribution. (**D**,**E**) Lipid droplet formation after neutralization of PAI-1 in conditioned media. (**D**) Fluorescence images of LDs. (**E**) Sum of LD areas. Data are presented as means ± SEM (n = 4~10). * *p* < 0.05, ** *p* < 0.01 vs. control.

As TCDD-induced adipocyte hypertrophy was absent in AhR LKO mice, we hypothesized that liver-derived factors mediate this effect. Previously, we reported that TCDD treatment induces PAI-1 expression and secretion from Hepa1c1c7 hepatoma cells, with secreted PAI-1 inducing apoptosis in pancreatic beta cells [27]. To test whether hepatic PAI-1 also promotes adipocyte hypertrophy, we treated 3T3-L1 adipocytes with either TCDD (100 pM) directly or with conditioned media from TCDD-treated Hepa1c1c7 cells. Both treatments increased lipid droplet size in 3T3-L1 cells (Figure 6A–C), suggesting that factors secreted by TCDD-treated hepatocytes enhance adipocyte lipid accumulation. To confirm the role of PAI-1, we neutralized it in the conditioned media using a specific antibody before treating 3T3-L1 cells. Neutralization of PAI-1 abolished the increase in lipid droplet size (Figure 6D,E), indicating that hepatic PAI-1 secretion promotes adipocyte hypertrophy following TCDD exposure.

### 2.7. TCDD-Induced PAI-1 Expression Impairs Hepatic tPA–ApoB Binding

**Figure 7:**
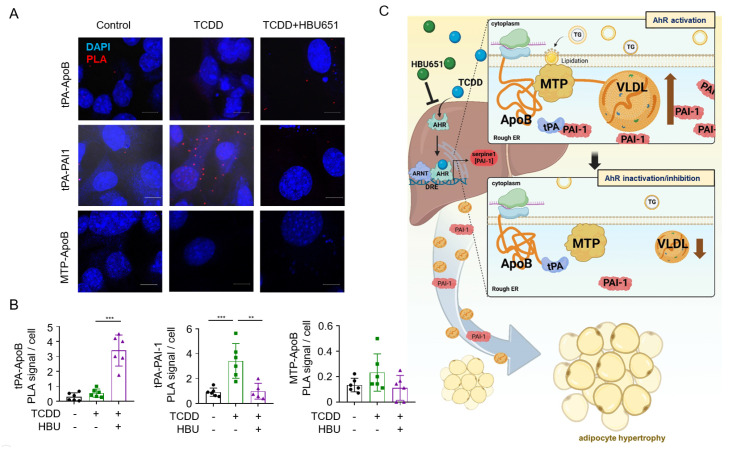
TCDD-mediated AhR activation disrupts tPA–ApoB interaction via PAI-1 induction in hepatocytes. (**A**) Proximity ligation assay (PLA) measuring tPA–PAI-1, tPA–ApoB, and MTP–ApoB interactions in Hepa1c1c7 cells. Scale bars = 10 μm. (**B**) Quantification of PLA signals using ImageJ. Data are presented as means ± SEM (n = 6). ** *p* < 0.01, *** *p* < 0.001. (**C**) Schematic illustrating how TCDD-induced AhR activation increases PAI-1 expression in hepatocytes, leading to displacement of tPA from ApoB and enhanced VLDL assembly and secretion. AhR inhibition by HBU651 restores tPA–ApoB interaction and reduces VLDL secretion. Additionally, increased hepatic PAI-1 is secreted into the bloodstream, contributing to adipocyte hypertrophy.

To determine whether AhR activation affects the interaction between tPA and ApoB, proximity ligation assays (PLA) were performed in Hepa1c1c7 cells. TCDD treatment resulted in a marked increase in tPA–PAI-1 interactions, suggesting enhanced binding of PAI-1 to tPA upon AhR activation (Figure 7A,B). Although the reduction in tPA-ApoB PLA signals with TCDD alone was modest, c-treatment with the AhR antagonist HBU651 significantly reversed the increased tPA-PAI-1 interaction and increased the tPA-ApoB interaction, indicating that these effects are at least partially AhR-dependent. In contrast, no significant changes were observed in MTP-ApoB across treatment conditions (Figure 7A,B). Quantification of PLA signals using ImageJ supported these findings (Figure 7B). These results suggest that TCDD-induced PAI-1 competes with ApoB for binding to tPA,, potentially modulating VLDL assembly (Figure 7C). However, this mechanism remains speculative and warrants further validation.

## 3. Discussion

In this study, we examined the role of hepatic AhR in mediating TCDD-induced metabolic alterations. A single injection of a moderate dose of TCDD induced lipid accumulation, inflammation, and mitochondrial dysfunction within 12 weeks. Notably, the liver-specific knockout of AhR not only mitigated hepatic steatosis but also attenuated adipocyte hypertrophy and macrophage infiltration in adipose tissues, suggesting that systemic lipid metabolism dysregulation is mediated by hepatic AhR activation.

While classical toxicological studies have associated TCDD exposure with cachexia and weight loss, especially under high-dose or chronic exposure conditions, recent research suggests a more nuanced picture depending on dose, timing, and dietary context. Our study, which employed a single moderate dose (10 μg/kg) of TCDD under a normal diet, revealed a progressive increase in adiposity, hepatic steatosis, and systemic inflammation over 12 weeks. These findings are consistent with emerging evidence indicating that low-dose or sub-chronic TCDD exposure—particularly when combined with a high-fat diet—can exert obesogenic effects rather than wasting phenotypes [10,30]. One possible explanation for these contrasting outcomes lies in the lipophilic nature of TCDD, which facilitates its long-term sequestration in adipose tissue. Such bioaccumulation may alter adipose tissue function and systemic lipid metabolism over time, even after circulating AhR ligand levels return to baseline. This delayed yet persistent metabolic dysregulation likely reflects a non-classical mode of TCDD toxicity that diverges from acute cachexia-inducing models and underscores the importance of dose and exposure timing in determining toxicological outcomes.

Serum AhR ligand levels in both WT and AhR LKO mice increased one week after TCDD injection but returned to baseline by 12 weeks (Figure 1D). However, hepatic Cyp1a1 expression remained elevated at 12 weeks (Figure 3C,D), indicating persistent AhR activation. This suggests that TCDD may accumulate in the fat tissues and exert prolonged effects, leading to lipid accumulation and inflammation in both hepatic and adipose tissues. Consistent with these findings, our previous studies showed elevated serum AhR ligand levels in patients with diabetes and metabolic syndrome [26,31]. Although serum AhR ligands were not detected at 12 weeks after a single TCDD injection in mice, elevated levels in patients likely reflect chronic repeated POP exposure, highlighting that even a single moderate-dose acute exposure can have harmful metabolic consequences after 12 weeks.

Previous studies have reported TCDD-induced hepatic steatosis and fibrosis in mice following repeated exposure [12,13,21,22]. Hepatic TG accumulation can arise from increased circulating fatty acid uptake, enhanced de novo lipogenesis, impaired TG secretion, or reduced β-oxidation of fatty acid. Fatty acid uptake is largely mediated by CD36, known as fatty acid translocase and a transcriptional target of nuclear receptors including AhR [32]. Transgenic mice engineered to express constitutively activated AhR showed up-regulation of CD36 and suppression of fatty acid oxidation and hepatic export of TGs [33]. TCDD has been shown to alter gene expression involved in lipid absorption and transport, lipolysis, macrophage recruitment, and β-oxidation, while suppressing de novo lipogenesis [13,21,34]. Our results are consistent with these mechanisms: TCDD injection significantly increased hepatic and serum TG levels and lipid droplet formation in WT mice, while reducing mitochondrial OCR, suggesting impaired fatty acid utilization leading to steatosis under AhR activation.

H2AX, an isoform of histone H2A, is phosphorylated in response to nuclear DNA damage and plays roles in DNA repair, recombination, and tumor suppression [35]. Beyond the nucleus, H2AX localizes to the mitochondrial outer membrane and interacts with TOM20 [36]. Loss of mitochondrial H2AX impairs TFAM expression, protein import, and mitochondrial function [29], and H2AX deficiency reduces OXPHOS complexes and increases susceptibility to mitochondrial toxins in vivo [37]. Thus, decreased TOM20 and H2AX levels observed in TCDD-injected livers may impair mitochondrial protein import and mtDNA maintenance, contributing to mitochondrial dysfunction. However, further studies are needed to elucidate these mechanisms.

Elimination of hepatic AhR significantly attenuated TCDD-induced hepatic steatosis, adipocyte hypertrophy, and macrophage infiltration. However, a modest increase in hepatic TG levels and reduction in mitochondrial respiratory capacity were still observed in AhR LKO mice following TCDD exposure. While AhR is a key transcriptional regulator of xenobiotic and lipid metabolism [38], these residual effects may stem from several non-mutually exclusive possibilities, such as incomplete gene deletion, activation of compensatory pathways, or alternative toxic mechanisms independent of canonical AhR signaling. For instance, TCDD has been shown to induce mitochondrial oxidative stress and reduce mitochondrial calcium content in an AhR-independent manner, contributing insulin resistance [39]. Although our findings underscore the central role of hepatic AhR in mediating the metabolic consequences of TCDD, we acknowledge that definitive evidence for AhR-independent toxicity in this model is limited. Therefore, the residual hepatic TG accumulation and mitochondrial impairment observed in AhR LKO mice should be interpreted with caution, while still highlighting the multifaceted and complex nature of TCDD-induced metabolic disturbances.

Interestingly, TCDD did not elevate serum TG levels in AhR LKO mice despite increased hepatic TG levels (Figure 3H,I), suggesting that circulating TGs contribute to adipose lipid accumulation and inflammation in an AhR-dependent manner. We previously reported PAI-1 secreted from hepatocytes following TCDD exposure contributes to pancreatic beta-cell apoptosis. PAI-1, an inflammatory adipokine and a recognized biomarker of metabolic syndrome [40,41], is closely associated with cardiovascular disease, hepatic steatosis, dyslipidemia, and adipose tissue dysfunction [42,43,44]. In this study, conditioned media from TCDD-treated Hepa1c1c7 cells increased lipid droplet size in differentiating 3T3-L1 adipocytes, while neutralization of PAI-1 abolished this effect, indicating that hepatic PAI-1 promotes adipocyte hypertrophy (Figure 6D,E). Mechanistically, proximity ligation assays revealed that TCDD enhanced PAI-1–tPA interactions, an effect reversed by AhR antagonist HBU651 (Figure 7). Although the reduction in tPA–apoB interaction following TCDD exposure was modest, co-treatment with HBU651 significantly increased this interaction. These findings suggest that PAI-1, upregulated via hepatic AhR activation, may compete with ApoB for tPA binding, thereby modulating ApoB lipidation and promoting VLDL assembly and biogenesis (Figure 7C). Although our data support a model in which hepatic PAI-1 disrupts tPA–ApoB interaction to enhance VLDL biogenesis, we acknowledge that tPA levels were not directly measured in vivo. Given the proposed role of tPA in this mechanism, future studies should assess hepatic tPA expression and activity in vivo, and validate the contribution of the PAI-1–tPA axis using hepatocyte-specific PAI-1 knockout models or pharmacological inhibition strategies. Furthermore, we used Hepa1c1c7 hepatoma and 3T3-L1-derived adipocytes for their reproducibility. However, these immortalized lines may not fully recapitulate primary cell physiology. Future studies using primary hepatocytes and adipocytes will be critical to validate these findings.

While the AhR is classically implicated in mediating the toxic effects of environmental pollutants such as TCDD, it is increasingly recognized that AhR also plays homeostatic roles in metabolism, immunity, and gut health. Several indole derivatives produced by gut microbiota—including indole-3-acetate and indole-3-aldehyde—act as endogenous AhR ligands and have been reported to exert beneficial effects by modulating lipid metabolism, improving insulin sensitivity, and attenuating adipose inflammation [45,46]. These findings emphasize the ligand-specific and tissue-context-dependent outcomes of AhR activation. In contrast to protective microbial ligands, persistent high-affinity xenobiotic ligands such as TCDD can drive maladaptive metabolic responses. Our observation that the AhR antagonist HBU651 reversed TCDD-induced PAI-1 upregulation and its downstream metabolic effects supports the concept that selective AhR inhibition can counteract environmental toxicant-induced dysfunction while potentially sparing beneficial endogenous AhR signaling. Therefore, the therapeutic strategy of targeting pathological AhR activation—without abolishing physiological signaling—may offer a balanced approach to managing pollutant-driven metabolic diseases.

In summary, our results demonstrate that a single TCDD exposure impairs mitochondrial protein expression and oxidative respiration, leading to hepatic steatosis in normal diet-fed mice. Hepatic AhR activation increases circulating TGs and induces PAI-1 secretion, promoting systemic lipid accumulation and inflammation. Although further research is needed to delineate the precise mechanisms and develop targeted interventions, these findings underscore the central role of hepatic AhR in metabolic homeostasis and suggest that modulation of hepatic AhR activity could be a promising strategy for treating obesity and related metabolic disorders.

## 4. Materials and Methods

### 4.1. Animal and Experimental Design

Wild-type C57BL/6J mice were purchased from Daehan Biolink (Eumsung, Republic of Korea), AhR^flox/flox^ (AhR^tm3.1Bra/J^, 006203) mice were purchased from the Jackson Laboratory (Bar Harbor, ME, USA) and albumin Cre (TgN(Alb-cre)Gto/J) mice were provided from Prof. Goo Taeg Oh (Ewha Womans University, Seoul, Republic of Korea) [27]. To generate liver-specific AhR knockout mice (AhR LKO), AhR^flox/flox^ mice were crossbred with albumin Cre mice. All mice were housed under controlled conditions with a 12 h light/dark cycle, 40% humidity, and a temperature of 23 °C, with ad libtum access to food and water. All animal care and experimental procedures conformed to the Principles of Laboratory Animal Care (NIH publication No. 85–23, revised 1985) and were approved by the Animal Research Ethics Committee of Kyung Hee University (KHUASP(SE)-23-031), in accordance with the institutional guidelines.

All experiments were designed and reported following the ARRIVE guidelines to ensure reproducibility and transparency. Sample sizes were determined based on prior studies to ensure adequate statistical power. Mice were randomly allocated to treatment groups, and investigators were blinded to group assignments during data collection and analysis where feasible. All efforts were made to minimize animal suffering and to reduce the number of animals used.

### 4.2. TCDD Injection

Wild-type C57BL/6 (8-week-old, male) mice were intraperitoneally injected with TCDD at doses of 0, 0.5, 1, 2, 5 or 10 μg/kg (n = 7 per group). The single dose regimen was selected based on previous reports modeling hepatic enzyme induction and tissue retention profiles within this dose range [47]. Body weight and food intake were monitored daily. Food intake was calculated by measuring the amount of food consumed per mouse. Blood samples were collected from the retro-orbital sinus at 1, 9, and 12 weeks post-injection, centrifuged, and stored at −80 °C until analysis. At 12 weeks post-injection, liver, epididymal white adipose tissue (WAT), and interscapular brown adipose tissues (BAT) were dissected. The right liver lobe and adipose tissues were fixed with 4% paraformaldehyde (PFA) for histological analysis, while the left liver lobe was used for mitochondrial analysis, and the median lobe was immediately frozen in liquid nitrogen and stored at −80 °C. In a second experiment, WT and AhR LKO mice were injected intraperitoneally with TCDD at doses of 0, 1, or 10 μg/kg. Blood and tissue samples were collected 12 weeks post-injection as described above.

### 4.3. Cell Culture and Treatment

Hepa1c1c7 (CRL-2026) and 3T3-L1 (CL0173) cells were obtained from the American Type Culture Collection (ATCC, Manassas, VA, USA). Hepa1c1c7 cells were cultured in Minimum Essential Medium Alpha (MEMα) supplemented with penicillin/streptomycin and 10% fetal bovine serum (FBS), while 3T3-L1 cells were cultured in Dulbecco’s Modified Eagle’s Medium (DMEM) containing penicillin/streptomycin and 10% bovine calf serum. All cells were maintained at 37 °C with 5% CO_2_. To collect conditioned media, Hepa1c1c7 cells were treated with TCDD for 24 h, followed by an additional 24 h incubation in fresh serum-free DMEM. The conditioned media were then collected and centrifuged at 1000× *g* for 10 min at 4 °C.

### 4.4. Measurement of AhR Ligands in Serum

Mouse serum was prepared by allowing blood to clot for 30 min at room temperature, followed by centrifugation at 1000× *g* for 20 min at 4 °C. Serum AhR ligands were measured using a cell-based AhR ligand activity (CALA) assay [24]. This assay employed dual luciferase reporters: pGL4.20-DRE-luc [Puro+], containing a dioxin-response element (DRE) fragment from the mouse Cyp1a1 promoter, and pGL4.76-mTK-Rluc [Hygro+], containing the minimal thymidine kinase promoter (Promega, Madison, WI, USA). Hepa1c1c7 cells were transfected with both reporters using Attractene (Qiagen, Hilden, Germany) according to the manufacturer’s instructions. Stable cell lines were established by culturing in 1 μg/mL puromycin and 200 ng/mL hygromycin B for at least 3 weeks. Reporter-transfected cells (5 × 10^4^ cells/well) were seeded into 96-well white plates one day prior to the assay. On the following day, media were replaced with MEMα containing 10% mouse serum samples. After 24 h, firefly and *Renilla* luciferase activities were measured using Dual-Glo^®^ (Promega) on a luminometer (Berthold Technologies, Bad Wildbad, Germany), with *Renilla* luciferase activity serving as the internal control.

### 4.5. Histochemistry

Liver and adipose tissues were fixed in 4% PFA, dehydrated, and embedded in paraffin. Paraffin blocks were sectioned at 5 μm thickness using a microtome. Sections underwent deparaffinization and rehydration through graded solvents, followed by hematoxylin and eosin (H&E; Abcam, Cambridge, UK) staining or immunostaining. For immunostaining, tissue sections were incubated with anti-F4/80 mouse IgG (1:200, Santa Cruz Biotechnology, Dallas, TX, USA), followed by Alexa Fluor™ 488-conjugated anti-mouse IgG (1:1000, Invitrogen, Waltham, MA, USA). Fluorescence images were acquired using a confocal microscope (LSM700, Carl Zeiss, Oberkochen, Germany).

For Oil Red O (ORO) staining, PFA-fixed liver and adipose tissues were rinsed with distilled water, immersed in 30% sucrose in PBS at 4 °C until sinking, and embedded in optimal cutting temperature (OCT) compound. Tissues were sectioned at 30 μm thickness using a cryostat (CM1860, Leica Biosystems, Wetzlar, Germany) and stained with ORO solution (Abcam). H&E- and ORO-stained sections were observed under an optical microscope (Olympus, Tokyo, Japan), and adipocyte perimeters were measured using ImageJ software (version 1.53e, http://imageJ.nih.gov/ij, accessed on 5 December 2023, National Institutes of Health, Bethesda, MD, USA).

### 4.6. Real Time Quantitative Reverse Transcription-PCR (qRT-PCR)

Total RNA was extracted from frozen liver tissues using TRIzol™ reagent (Invitrogen). cDNA was synthesized from 2 μg of total RNA using MMLV reverse transcriptase (Promega) with 10 pM oligo(dT) primers and 25 mM dNTPs. qRT-PCR was performed using AMPIGENE^®^ qPCR Green Lo-ROX (Enzo Life Sciences, Farmingdale, NY, USA) on a Rotor-Gene Q (Qiagen) platform under the following cycling conditions: 95 °C for 2 min, followed by 40 cycles of 95 °C for 5 s and 60 °C for 30 s. Relative gene expression levels were calculated using the 2^−ΔΔCt^ method [48] and normalized to 18S rRNA. Primer sequences used were: AhR (5′-ATCGCCACTCAGAGA-CCACT-3′ and 5′-AGGGCTGGAGATCTCGTACA-3′) Cyp1a1 (5′-TCCGGCATTCATCCTT-CGTC-3′ and 5′-ACAGTTCCCGGTCATGGTTA-3′) 18S rRNA (5′-GAGCGAAAGCATTTG-CCAAG-3′ and 5′-GGCATCGTTTATGGTCGGAA-3′).

### 4.7. Western Blot Analysis of Mitochondrial Proteins

To isolate mitochondria, liver samples were homogenized in mitochondria isolation buffer (250 mM sucrose, 25 mM Tris-HCl pH 7.4, 1 mM EDTA) containing protease and phosphatase inhibitors [29]. Homogenates were centrifuged at 1000× *g* for 10 min at 4 °C, and the supernatants were further centrifuged at 7500× *g* for 10 min at 4 °C to pellet mitochondria. The mitochondrial pellets were resuspended in lysis buffer (50 mM Tris-HCl pH 7.4, 150 mM NaCl, 1% Triton X-100, 10% glycerol) containing protease and phosphatase inhibitors, and centrifuged at 16,000× *g* for 10 min at 4 °C to remove debris. Protein concentrations were measured using a BCA Protein Assay Kit. Mitochondrial lysates were separated on 10% or 15% SDS-PAGE gels, and transferred to PVDF membranes. Membranes were incubated with primary antibodies against mitochondrial proteins of NDUFA9 (1:2000, 459100, Thermo Fisher Scientific, Waltham, MA, USA), COX1 (1:2000, Cell Signaling Technology, Danvers, MA, USA), anti-complex II cocktail (SDHA, SDHB, ATP5A, 1:2000, ab110410, Abcam), UQCRC2, (Abcam), HSP60 (1:2000, sc-13115 Santa Cruz Biotechnology, Dallas, TX, USA, TOM20 (1:2000, sc-17764 Santa Cruz), TFAM (1:2000, sc-23588, Santa Cruz Biotechnology), and H2AX (1:2000, R&D Systems, Minneapolis, MN, USA) [29] in Tris-buffered saline with 0.1% Tween 20 (TBST) containing 3% BSA. Bands on the PVDF membrane were detected by G:BOX Chemi XL1.4 (Syngene, Frederick, MD, USA) using EzWestLumi plus (ATTO, Tokyo, Japan) and band intensities were quantified using ImageJ program. All uncropped scans of Western blots are shown in Supplementary Information.

### 4.8. Triglyceride Assay

Triglyceride (TG) levels in mouse serum and liver were measured using a Triglyceride Colorimetric Assay Kit (Cayman Chemical, Ann Arbor, MI, USA) according to the manufacturer’s instructions. Liver samples were homogenized in NP40 Substitute Assay Reagent provided in the kit and centrifuged at 10,000× *g* for 10 min at 4 °C. Serum and liver lysates were incubated with the enzyme mixture solution at 37 °C for 30 min, and absorbance was measured at 540 nm using a microplate reader (Versamax, Molecular Devices, San Jose, CA, USA).

### 4.9. Transmission Electron Microscopy

Liver samples were fixed in 2.5% glutaraldehyde in 0.1 M phosphate buffer (pH 7.0) at 4 °C overnight, washed with the same buffer, and post-fixed with 1% osmium tetroxide in 0.1 M phosphate buffer (pH 7.0) for 2 h at 4 °C. Samples were then dehydrated through graded ethanol and embedded in Spurr’s resin. Ultrathin sections were mounted on copper grids and observed using a transmission electron microscope (JEM-2100, JEOL, Tokyo, Japan).

### 4.10. Oxygen Consumption Rate

Seahorse XF24 cell culture microplates were calibrated with XF calibrant (Agilent Technologies, Santa Clara, CA, USA) at 37 °C overnight in a non-CO_2_ incubator. Isolated mitochondria (10 μg/well) were loaded onto the plate, and oxygen consumption rate (OCR) was measured every 7 min for 100 min using a Seahorse XF-24 Analyzer (Agilent Technologies, Santa Clara, CA, USA) [49,50]. During the measurement, ADP (2 mM), oligomycin (1 μM), carbonyl cyanide-4-(trifluoromethoxy) phenylhydrazone (FCCP, 1 μM), and rotenone (1 μM) were sequentially added. Basal respiration was calculated by subtracting OCR without ADP from OCR with ADP. ATP turnover and respiratory capacity were calculated by subtracting oligomycin-inhibited OCR or rotenone-inhibited OCR from basal respiration or FCCP-stimulated OCR, respectively.

### 4.11. Adipogenesis Assay

3T3-L1 cells (3 × 10^4^ cells/well) were seeded in 96-well black plates. The next day, differentiation was induced using DMEM containing 10% FBS and a hormone cocktail (Abcam) consisting of 500 μM isobutylmethylxanthine, 1 μM dexamethasone, and 10 μg/mL insulin. After 2 days, induction media were replaced with DMEM containing 10% FBS and insulin, and cells were further incubated for 6 days with media changes every 2 days. At the end of differentiation, cells were treated with TCDD or conditioned media (containing 10% FBS) for 24 h. For neutralization experiments, conditioned media were pre-incubated with a PAI-1 antibody (1:30, Abcam) for 2 h at room temperature. After treatments, cells were washed with DPBS, fixed with 2% PFA for 10 min at room temperature. Lipid droplets were stained with Bodipy (5 μg/mL, Invitrogen) for 60 min or Nile red (1 μg/mL, Sigma-Aldrich, St. Louis, MO, USA) for 30 min at room temperature. Fluorescence images were acquired using a digital cell imaging system (iRiS™, Logos Biosystems, Anyang, Republic of Korea), and lipid droplet diameters were measured using ImageJ.

### 4.12. Proximity Ligation Assay

Protein–protein interactions were assessed using a proximity ligation assay (PLA; Sigma-Aldrich, St. Louis, MO, USA, DUO92101). Cells (1 × 10^4^ cells/well) were cultured for 24 h on 12 mm coverslips in 8-well chamber slides (SPL Life Sciences, Pocheon, Republic of Korea), fixed with 4% PFA for 15 min at room temperature, and blocked with 0.1% Triton X-100 in PBS containing 5% BSA for 1 h. Samples were incubated overnight at 4 °C with pairs of primary antibodies: rabbit anti-PAI-1 (1:200; Abcam), with mouse anti-MTP (1:200; Santa Cruz) or mouse anti-tPA (1:200; Abcam) with rabbit anti-ApoB (1:200; Proteintech, Rosemont, IL, USA), diluted in 0.1% Triton X-100 in PBS with 3% BSA. After washing, cells were incubated with PLUS and MINUS PLA probes for 1 h at 37 °C, followed by ligation (30 min at 37 °C) and amplification (100 min at 37 °C), according to the manufacturer’s instructions. Samples were washed, mounted with DAPI-containing medium (Vector Laboratories, Burlingame, CA, USA), and visualized using a laser scanning confocal microscope (Carl Zeiss, Oberkochen, Germany).

### 4.13. Statistical Analysis

All statistical analyses were performed using GraphPad Prism (GraphPad Software, Prism 8.0.1, San Diego, CA, USA). Data are presented as means ± standard error of the mean (SEM). Statistical differences between two groups were evaluated using unpaired Student’s *t*-tests, and *p*-values < 0.05 were considered statistically significant. Statistical significance is denoted in the figure legends as follows: * *p* < 0.05, ** *p* < 0.01, *** *p* < 0.001, and in some cases, comparisons between genotypes are indicated using number signs (#, ##, ###). The number of biological replicates (n) is specified in each figure legend.

## 5. Conclusions

This study demonstrates that a single moderate-dose TCDD exposure under acute condition impairs mitochondrial protein expression and oxidative respiration, leading to hepatic steatosis in normal diet-fed mice. Hepatic AhR activation increases circulating triglycerides and induces PAI-1 secretion, promoting inflammation and obesity. Additionally, TCDD-induced hepatic PAI-1 disrupts tPA–ApoB interactions, enhancing VLDL assembly. These findings reveal that hepatocyte-derived PAI-1, upregulated via AhR activation, contributes to systemic lipid accumulation and metabolic dysfunction. Overall, these findings extend the understanding of environmental pollutant-induced metabolic disorders and identify the hepatic AhR–PAI-1 axis as a potential therapeutic target for obesity and related metabolic diseases.

## Data Availability

The original contributions presented in this study are included in the article. Further inquiries can be directed to the corresponding authors.

## Supplementary Materials

The following supporting information can be downloaded at: https://www.mdpi.com/article/10.3390/ijms26178452/s1.

## Abbreviations

The following abbreviations are used in this manuscript:

AhR	Aryl hydrocarbon receptor
AhRL	Aryl hydrocarbon receptor ligand
ApoB	apolipoprotein B
BAT	Brown adipose tissue
CALA	cell-based AhR ligand activity
COX1	cytochrome C oxidase subunit 1
LD	Lipid droplet
LKO	Liver specific knock-out
MTP	Microsomal triglyceride transfer protein
OCR	oxygen consumption rate
ORO	Oil red O
OXPHOS	oxidative phosphorylation
PAI-1	plasminogen activator inhibitor-1
PLA	Proximity ligation assay
POPs,	persistent organic pollutants
TCDD	2,3,7,8-tetrachlorodi-benzodioxin
TFAM	transcription factor A, mitochondrial
TG	triglyceride
tPA	tissue-type plasminogen activator
TOM20	translocase outer mitochondrial membrane 20
VLDL	very-low-density lipoprotein
WAT	white adipose tissue
WT	wild type
