# Supplementary material for: Hepatic AhR Activation by TCDD Induces Obesity and Steatosis via Hepatic Plasminogen Activator Inhibitor-1 (PAI-1)
Source: Int J Mol Sci. 2025 Aug 30;26(17):8452. doi: 10.3390/ijms26178452 (PMC12428968; doi:10.3390/ijms26178452)
Supplement: Supplementary file 1 [file ijms-26-08452-s001.zip › ijms-3777914-supplementary.pdf]

Supplementary Information

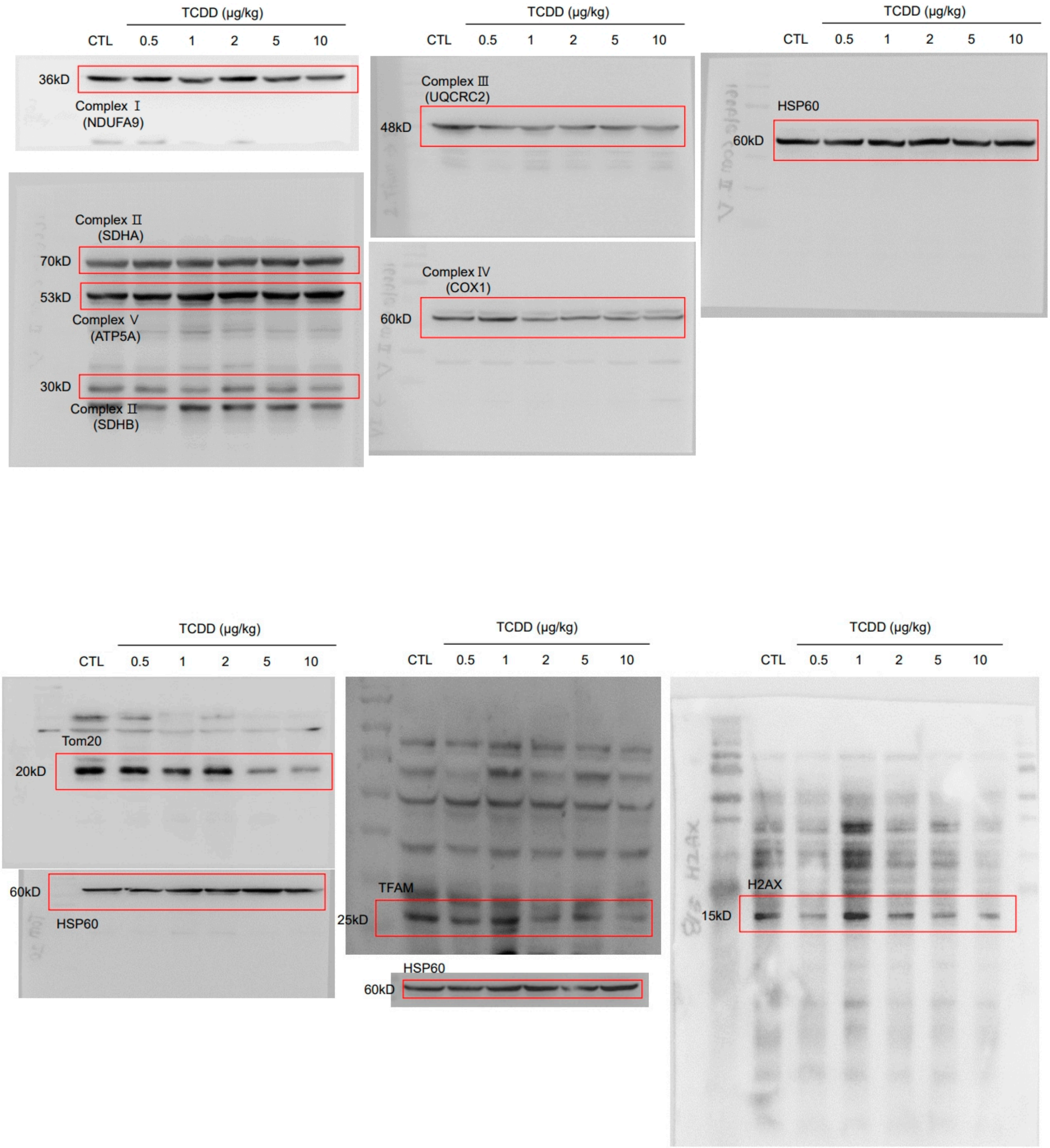

Supplementary Fig. S1. Uncropped original Western blot images corresponding to Figure 2. Western blot analysis of mitochondrial oxidative phosphorylation (OXPHOS) complex subunits was performed on liver tissues from mice exposed to TCDD. Representative subunits from complexes I (NDUFA9), II (SDHA, SDHB), III (UQCRC2), IV (COX1), and V (ATP5A), Tom20, TFAM, and H2AX were analyzed. HSP60 was used as a mitochondrial loading control. Full-length blot images are shown for transparency and validation of quantification.

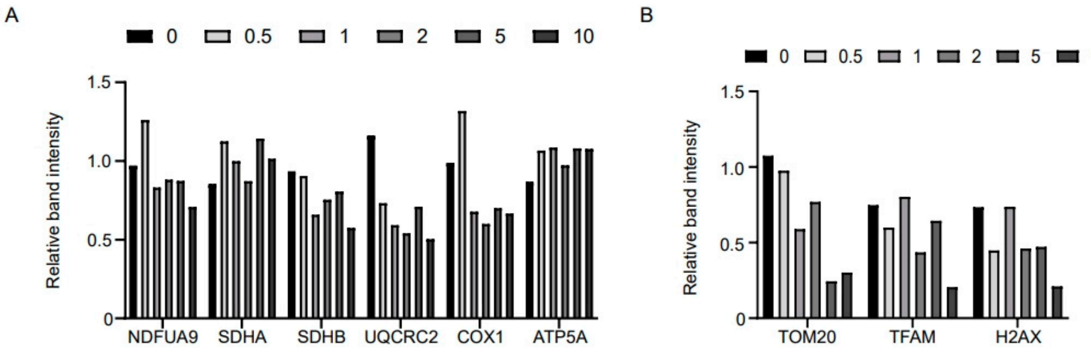

Supplementary Fig. S2. Quantification of mitochondrial OXPHOS complexes and mitochondrial markers in WT and AhR LKO mice (related to Figure 2).

Densitometric analysis of Western blots for representative subunits of mitochondrial oxidative phosphorylation (OXPHOS) complexes in liver tissues from WT and AhR LKO mice exposed to TCDD (0, 0.5, 1, 2, 5, and 10 μg/kg). (A) Relative protein levels of OXPHOS subunits: Complex I (NDUFA9), Complex II (SDHA, SDHB), Complex III (UQCRC2), Complex IV (COX1), and Complex V (ATP5A). (B) Relative protein levels of mitochondrial markers (TOM20, TFAM, H2AX). HSP60 was used as a mitochondrial loading control. Data are shown as mean ± SEM (n=6) of densitometric values normalized to HSP60**.**

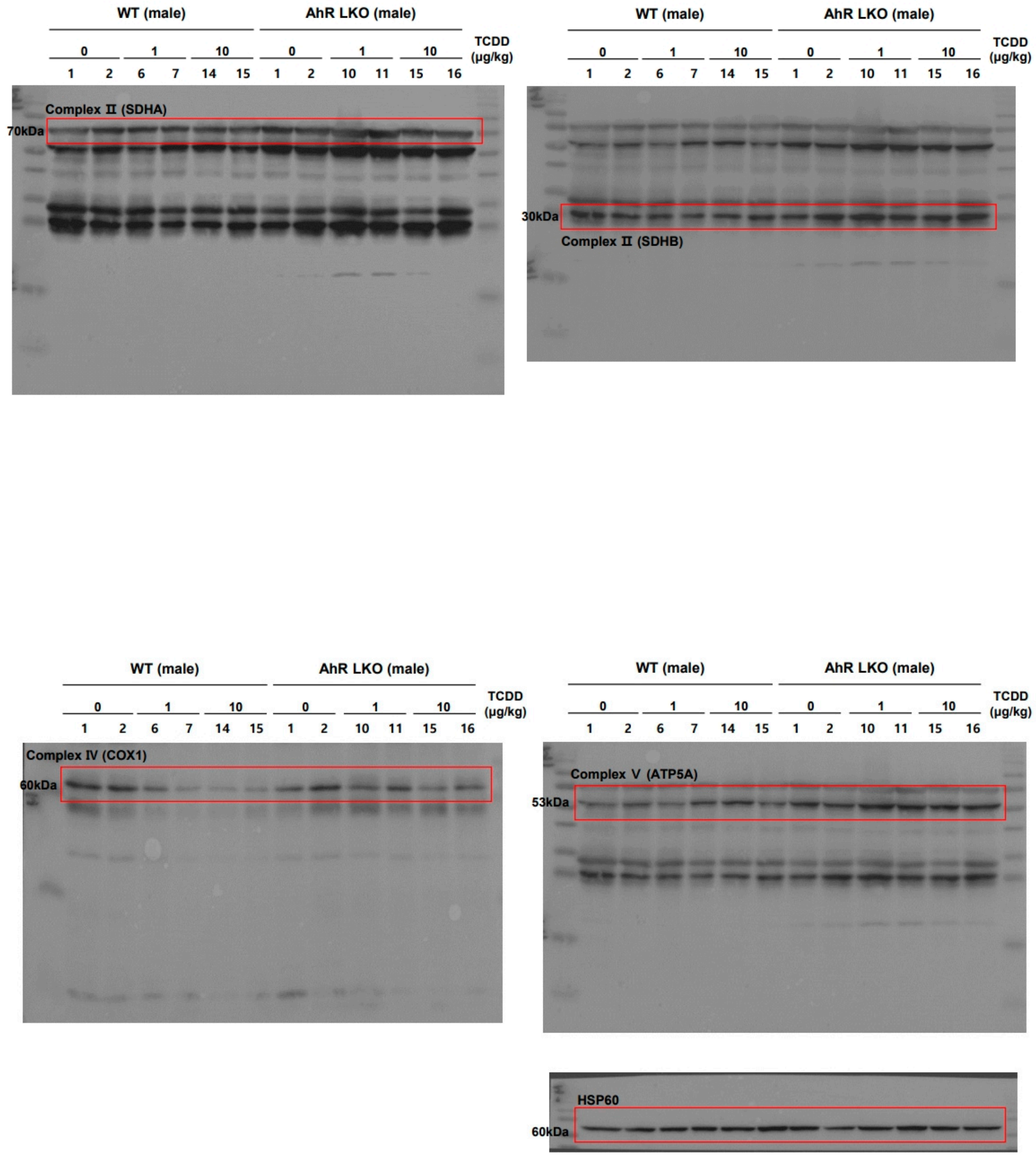

Supplementary Fig. S3. Uncropped original Western blot images corresponding to Figure 5B. Western blot analysis was performed on liver tissues from WT and AhR LKO mice exposed to TCDD. Representative subunits from Complex II (SDHA and SDHB), Complex IV (COX1) and Complex V (ATP5A) were analyzed. HSP60 was used as a mitochondrial loading control. Full-length blot images are shown for transparency and validation of quantification.

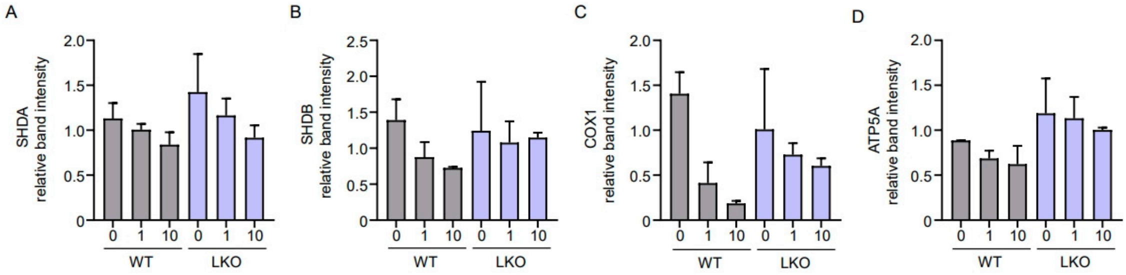

Supplementary Fig. S4. Quantification of individual mitochondrial OXPHOS complex subunits in WT and AhR LKO mice (related to Figure 5B).

Densitometric analysis of Western blots for representative OXPHOS subunits in liver tissues from WT and AhR LKO mice exposed to TCDD at the indicated doses (0 and 10 μg/kg). (A) Complex II subunit SDHA. (B) Complex II subunit SDHB. (C) Complex IV subunit COX1. (D) Complex V subunit ATP5A. HSP60 was used as a mitochondrial loading control. Data are shown as mean ± SEM (n=6) of densitometric values normalized to HSP60.
